# Cholecystoduodenal Fistula Evading Imaging and Endoscopic Retrograde Cholangiopancreatography: A Case Report

**DOI:** 10.7759/cureus.20049

**Published:** 2021-11-30

**Authors:** Charles K Lee, Darren N Ramcharan, Kayla L Alaimo, Veronica Velez, Anika E Risden, Dhadon H Klein, Osbaldo Garcia, Vaidehi Joshi, Juaquito M Jorge

**Affiliations:** 1 Medicine, Saint James School of Medicine, Park Ridge, USA; 2 Surgery, West Suburban Medical Center, Oak Park, USA; 3 Internal Medicine, Saint James School of Medicine, Park Ridge, USA; 4 Medicine, Avalon University School of Medicine, Willemstad, CUW; 5 General and Bariatric Surgery, West Suburban Medical Center, Oak Park, USA

**Keywords:** endoscopic retrograde cholangiopancreatography, ercp, internal biliary fistula, cholecystectomy, chronic cholelithiasis, cholecystoduodenal fistula

## Abstract

Cholecystoduodenal fistulas are a type of internal biliary fistula that occur due to chronic inflammation of the gallbladder/biliary tree; if left untreated, perforation and necrosis can occur. Cholecystoduodenal fistulas are often difficult to diagnose due to their non-specific signs and symptoms. Since the widespread use of techniques such as magnetic resonance cholangiopancreatography and imaging modalities such as computed tomography, the frequency of reports describing intraoperative cholecystoduodenal fistula has reduced dramatically.

Here, we report the case of a 54-year-old female who presented with a two-day history of non-radiating epigastric abdominal pain, initially diagnosed with acute cholecystitis and choledocholithiasis. Upon undergoing laparoscopic cholecystectomy, she was found to have extensive fibrosis of the gallbladder, adhesions, and an impacted gallstone in the wall of the gallbladder. Imaging and endoscopic retrograde cholangiopancreatography performed prior to surgery did not detect a cholecystoduodenal fistula that was discovered intraoperatively. She was treated successfully with laparoscopic cholecystectomy and repair of the duodenum.

## Introduction

A fistula is an abnormal connection between two organs or vessels that are normally not connected. They are named based on the involved organs or vessels (e.g., arteriovenous fistula, cholecystoduodenal fistula, etc.). The most common type of cholecystoenteric fistula is the cholecystoduodenal type, followed by cholecystocolonic (gallbladder to colon) and cholecystogastric fistula (gallbladder to stomach) [1,2]. Cholecystoduodenal fistulas, as the name suggests, are connections between the gallbladder and duodenum.

Cholecystoduodenal fistulas occur due to chronic inflammation of the gallbladder/biliary tree and are associated with gallstones, malignancy, cholecystectomy, common bile duct exploration, or penetrating trauma [1]. If left untreated, perforation and necrosis can occur [1]. Cholecystoduodenal fistulas are often difficult to diagnose due to their non-specific signs and symptoms [3-6], which can include abdominal pain, fever, nausea, vomiting, flatulence, fat intolerance, diarrhea, and weight loss, all of which are seen across many gastrointestinal pathologies. Patients often have a mean age of presentation in the fifth or sixth decade of life [2,4,5]. Cholecystoduodenal fistulas are often a complication of chronic cholelithiasis, typically inflammation is already present, and patients are often in poor condition, necessitating a decision between immediate/emergent treatment or waiting for natural closure [3]. Prior to the widespread use of techniques such as magnetic resonance cholangiopancreatography (MRCP) and imaging modalities such as computed tomography (CT), the majority of cholecystoduodenal fistulas were intraoperative findings [5,6]. Since these modalities have become commonplace, the frequency of reports describing intraoperative cholecystoduodenal fistula has reduced dramatically. To our knowledge, our report is one of only two such reports in the literature within the past decade.

Our case report discusses a 54-year-old female with a medical history of biliary stricture and choledocholithiasis presenting with weakness associated with epigastric and back pain. Ultrasound findings at the time of presentation were suggestive of cholecystitis, choledocholithiasis, and a positive Murphy’s sign.

## Case presentation

Chief complaint

A 54-year-old female presented to the emergency department complaining of weakness for two days with associated abdominal epigastric and back pain with bilateral lower extremity discomfort.

History of present illness

The patient’s weakness and epigastric pain began two days prior. The epigastric pain did not radiate and was localized to the periumbilical region. The pain was unchanged by food and not associated with any diarrhea, vomiting, fever, chills, headache, chest pain, or cough.

Medical history

The patient’s medical history included diabetes mellitus, hypertension, and a tracheostomy due to laryngeal cancer, as well as choledocholithiasis in 2017 which was treated with stone extraction and biliary stent placement at another facility. The patient was unable to specify the facility where her stone extraction and stent placement took place, nor the specific procedure used for stone extraction. The patient had also presented to the emergency department two months before her surgical intervention with complaints of sharp bilateral flank pain with one episode of nausea and vomiting and endorsed a history of kidney stones. A CT imaging of the abdomen performed at that time (images unavailable at the time of this report) ruled out nephrolithiasis or ureterolithiasis but revealed a biliary stent and mild pneumobilia.

Examination and investigations

Physical examination at the time of presentation revealed icteric sclera, jaundice, epigastric tenderness with mild guarding, and a positive Murphy’s sign.

Initial laboratory findings were as follows: hemoglobin 9.8 g/dL, white blood cell count 12,300 cells/µL, alkaline phosphatase 534 IU/L, alanine aminotransferase (ALT) 27 IU/L, aspartate aminotransferase (AST) 25 IU/L, and total bilirubin 6.4 mg/dL.

**Figure 1:**
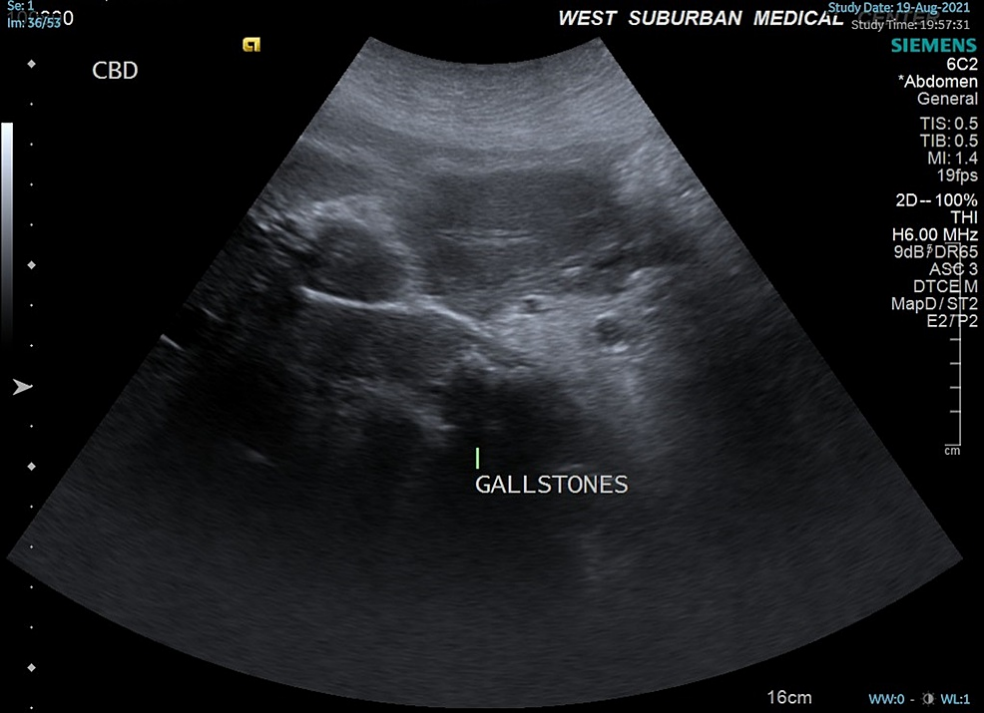
Ultrasound image showing multiple gallstones.

Ultrasound performed at the time of presentation (Figure 1) revealed multiple gallstones with gallbladder wall thickening, pericholecystic fluid suggesting cholecystitis, and choledocholithiasis with common bile duct dilation to 2.1-2.3 cm. A biliary stent was not observed at this time.

**Figure 2:**
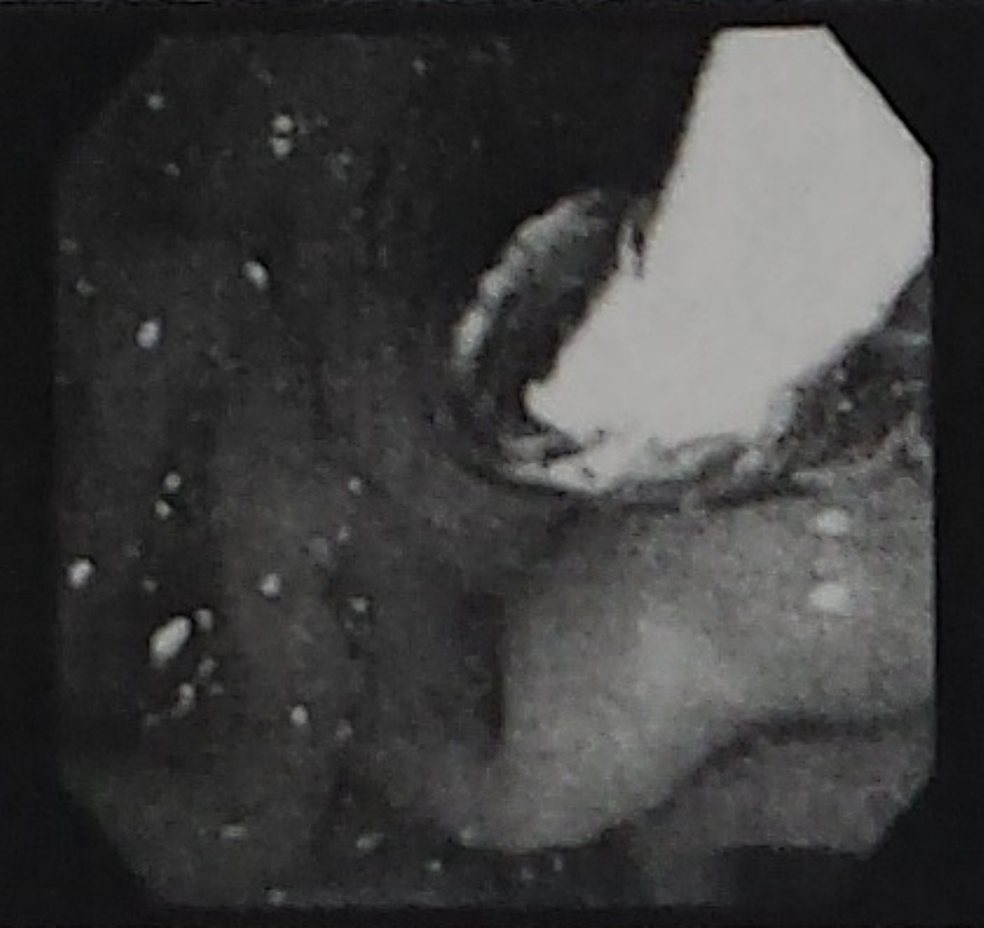
ERCP image showing pre-obstructive dilation. ERCP: endoscopic retrograde cholangiopancreatography

**Figure 3:**
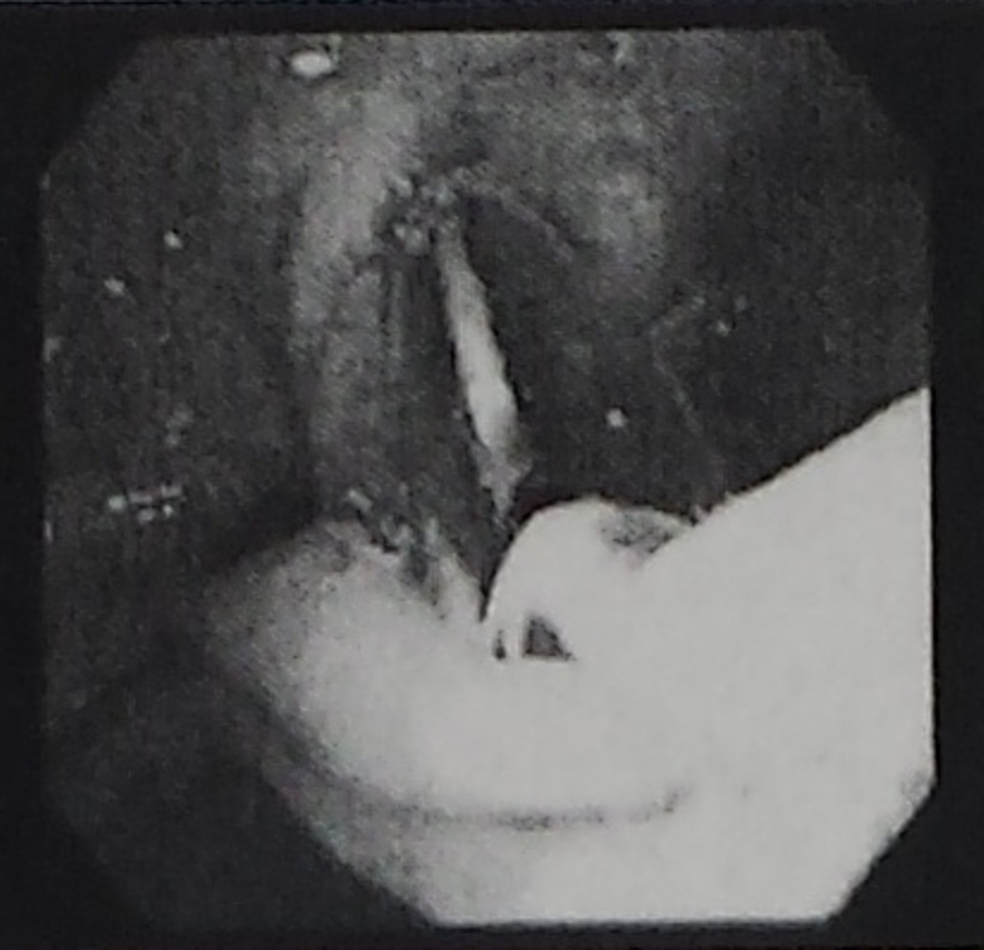
ERCP image showing a benign-appearing single smooth stricture 1 cm in length. ERCP: endoscopic retrograde cholangiopancreatography

**Figure 4:**
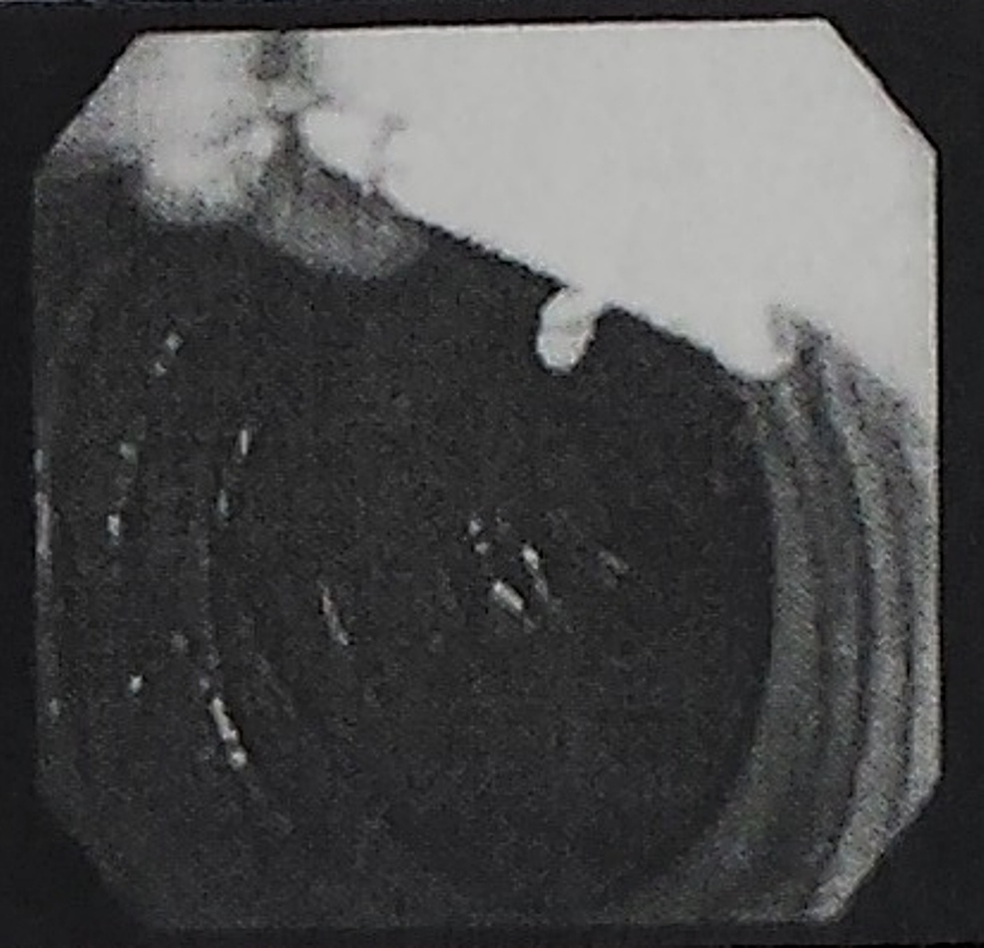
ERCP imaging showing placement of a 5-cm-long 10-French biliary stent. ERCP: endoscopic retrograde cholangiopancreatography

Three days prior to her laparoscopic surgery, our patient underwent endoscopic retrograde cholangiopancreatography (ERCP) for common bile duct stone clearance, sphincter dilation with stent placement, and sphincterotomy. Among the findings was a biliary stricture with pre-obstructive dilation (Figures 2, 3). A 5-cm-long 10-French biliary stent was also placed (Figure 4). Cholecystoduodenal fistula was not among the findings of the ERCP, and prior stenting was not observed at this time.

Preoperative diagnosis and treatment

**Figure 5:**
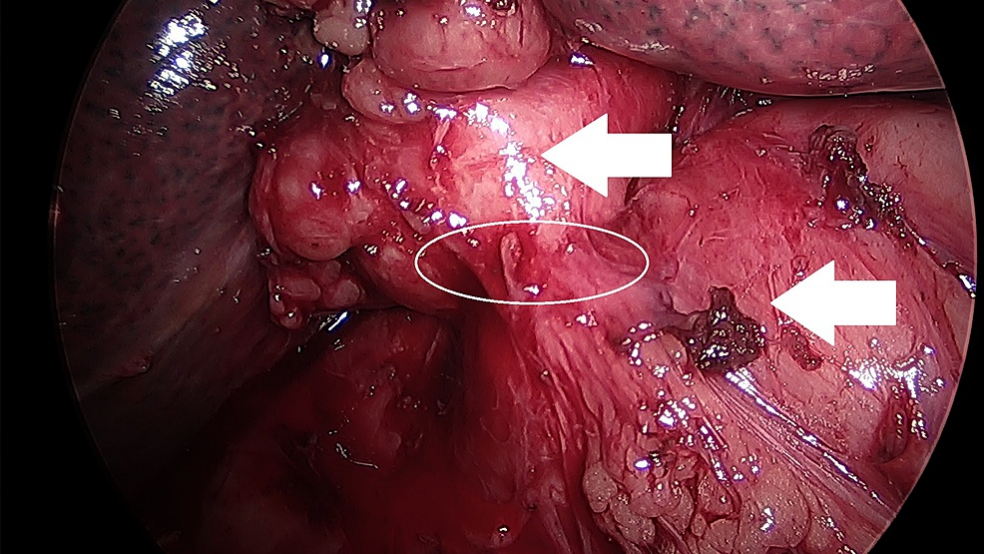
Intraoperative photograph of the patient’s cholecystoduodenal fistula. The top arrow indicates the inflamed gallbladder, lower arrow indicates the duodenum. Cholecystoduodenal fistula is circled.

The preoperative diagnosis was acute cholecystitis and choledocholithiasis. ERCP with common bile duct stone clearance, sphincter dilation with stent replacement, and sphincterotomy were performed three days prior to the surgery. The patient then underwent laparoscopic cholecystectomy. Operative findings revealed extensive fibrosis and acute chronic inflammation of the gallbladder with dense adhesions to surrounding structures, requiring dissection of the colon, duodenum, and omentum. Colotomy repair of the transverse colon was needed following the dissection due to 1-2 cm of full-thickness injury. No enterotomy was noted at the time of gallbladder dissection. Operative findings further revealed a gallstone impacted in and adherent to the wall of the gallbladder and a fistula opening into the duodenum. The gallstone was removed and fistula repair was completed with an omental patch overlay (Graham patch repair) of the duodenum. Images of the cholecystoduodenal fistula were taken intraoperatively (Figure 5).

Postoperative diagnosis

The postoperative diagnosis was acute cholecystitis, choledocholithiasis, cholecystoduodenal fistula, and an enlarged fatty liver.

Outcome/progress

The patient’s postoperative course was uneventful. The patient’s alkaline phosphatase, total bilirubin, and white blood cells had improved to 399 IU/L, 1.7 mg/dL, and 11,600 cells/µL, respectively. Three days after the surgery, a small bowel follow-through showed no evidence of duodenal leakage, contrast extravasation, or small bowel obstruction. The patient was discharged home five days after the surgery on a clear liquid diet and is currently being followed in an outpatient setting.

## Discussion

Cholecystoduodenal fistula, a type of internal biliary fistula, is a late and relatively rare complication of chronic cholelithiasis [3]. Several studies have suggested that the overall incidence of internal biliary fistulas (of which cholecystoduodenal fistula is a subtype) is between 0.15% and 5% [4,7], of which nearly all cases have been reported to occur in patients over the age of 50 [2-6,8,9]. Before the widespread use of techniques such as ERCP, MRCP, and CT, the majority of cholecystoduodenal fistulas were intraoperative findings [5,6]. After 2006, there is only one other case of cholecystoduodenal fistula discovered intraoperatively reported among existing literature [10].

An earlier case study proposed that the recent lack of literature reporting the intraoperative discovery of these types of fistulas may be because of the discovery of such fistulas during imaging or procedures such as ERCP or MRCP, which often precede surgical treatment of cholelithiasis or cholecystitis [11]. A 2006 study reviewing a single center’s data from January 1997 to June 2003 indicated that most cholecystoduodenal fistulas in the dataset were incidental findings during surgery [9]. Since then, the only other report of an intraoperative cholecystoduodenal fistula diagnosis was published in a 2019 paper in the Thai Journal of Surgery reporting on an incidental finding during elective laparoscopic cholecystectomy [10].

To our knowledge, sensitivity and specificity data for the detection of cholecystoduodenal fistulas by ERCP, MRCP, CT, and ultrasound have not been reported. However, the sensitivity and specificity data for the detection of related conditions such as gallstone ileus and Mirizzi syndrome (another type of internal biliary fistula, cholecystocholedochal fistula, is classified as type 2 Mirizzi syndrome [12,13]) have been reported. CT has been reported to be up to 93% sensitive and 100% specific for the detection of gallstone ileus [14], and ERCP has been reported to have a mean sensitivity of 76.2% for Mirizzi syndrome [15-17]; indeed, a 2013 study suggested a 100% sensitivity rate of ERCP for Mirizzi syndrome [18].

Our case demonstrates that ERCP and/or prior imaging may not always detect cholecystoduodenal fistulas, even though advances in imaging may have greatly improved their detection prior to surgery. Further, our case also demonstrates that incidental findings of cholecystoduodenal fistulas can be satisfactorily treated laparoscopically, as has been reported elsewhere [7,9,10,19].

## Conclusions

Due to the various and non-specific presentations of cholecystoduodenal fistula as a sequela of chronic cholelithiasis, cholecystoduodenal fistulas prior to the early 2000s were usually diagnosed intraoperatively. Although the advancement and widespread use of imaging modalities such as ERCP and MRCP have allowed the detection of cholecystoduodenal fistulas prior to surgery, our case demonstrates that they may still evade detection and be discovered incidentally during surgery. A high degree of clinical suspicion for cholecystoduodenal fistula should be retained for patients who present with gallstones, a positive Murphy’s sign, and choledocholithiasis with a medical history of biliary disease.
